# Determination of 4-tert-butyl pyrocatechol content in styrene with high accuracy using a chromatographic device under safe conditions: a laboratory study

**DOI:** 10.1038/s41598-025-99660-y

**Published:** 2025-04-30

**Authors:** Mohammad Heydari, Mohammad Reza Ghavidel

**Affiliations:** Researcher of EB/SM Laboratory, EB/SM Unit, Pars Petrochemical Company, Asalouyeh, Bushehr Iran

**Keywords:** 4 tert-butylpyrocatechol, Gas chromatography, Pars petrochemical company, Styrene, UV visible, Environmental chemistry, Environmental sciences, Environmental social sciences

## Abstract

A novel analytical method was developed for the determination of 4-tert-butylpyrocatechol (TBC) in styrene. Various measurement methods were compared. TBCs were analyzed using a straightforward method, which had significantly less contamination, and no additional chemicals were added to the solution. A non-polar column and temperature programming was then followed by an analysis and quantification of the final product to achieve this objective. In the range of 5 to 40 mg/kg (R^2^ ≥ 0.9999), the TBC peak area demonstrated notable linearity. Using the same technique, the obtained results were compared to TBC-containing real, standard, and proficiency test (PT) samples. This method showed the limit of detection (LOD) of 0.04–0.56 mg/kg and the limit of quantification (LOQ) of 0.15–1.96 mg/kg. The relative standard deviation (n = 15) was statistically lower than 10%. With fewer reagent requirements, contamination, and exposure to styrene due to carcinogenicity, this technique enables easy analysis of large amounts of samples. Therefore, it is possible to use it as a cutting-edge and creative method to measure the TBC content of styrene, particularly during routine analysis.

## Introduction

### Exposure to styrene

Large industries, particularly petrochemical plants, are significant sources of volatile organic compounds (VOCs)^1,2^. Exposure to VOCs can lead to a range of acute and chronic health issues, including cancer, nervous system disorders, and reduced lung function. Annually, millions fall ill or die due to chemical exposure^3^. Among these chemicals, styrene is widely used in producing plastics, rubber, polyester resins, fiberglass, toys, and household items^4–6^. Styrene, an aromatic hydrocarbon derived from benzene, is colorless, sweet-smelling, and evaporates quickly, with the chemical formula C_6_H_5_CH=CH_2_ or C_8_H_8_^7,8^. Occupational settings are the primary source of styrene exposure^9^. It enters the body through inhalation or skin contact, with inhalation being the dominant route in workplaces. Once absorbed, styrene enters the bloodstream and spreads throughout the body^10^. Styrene monomers act as central nervous system depressants, causing acute effects like fatigue, headaches, attention deficits, and hearing loss^11,12^. Styrene monomers act as central nervous system depressants, causing acute effects like fatigue, headaches, attention deficits, and hearing loss^13–15^.

### The significance of monitoring styrene

Styrene monomer is essential for producing polymer products like electrical insulators but is prone to undesirable polymerization due to heat, light, or transportation^4,16,17^. To prevent this, inhibitors such as 4-tert-butylpyrocatechol (TBC) are added^18–20^. Direct analysis of styrene samples is challenging due to low TBC levels and styrene’s carcinogenic nature. Gas chromatography with flame ionization detection (GC/FID) addresses this by using small sample volumes (1 µL) and avoiding hazardous reagents, ensuring safer and greener analysis^21,22^.

### Research purposes

In this research, a chromatographic method for determining TBC in styrene is reported. The conditions for measuring TBC in styrene are much simpler than the methods that have been reported so far. In this study, the comparison results of chromatography with an alkaline colorimetric method to determine TBC in styrene are presented and discussed. On the other hand, to reduce the exposure to styrene, especially in the laboratory environment during its analysis, the appropriate method with the least amount of contamination should be chosen. A GC/FID method for TBC determination was developed in this research for the first time.

## Materials and methods

### Chemicals, reagents, and materials

Analytical grade TBC was obtained from Fluka (Switzerland), and pure styrene with a grade of 99.99% was stabilized with TBC and obtained from Pars Petrochemical Company. Toluene, methanol, and n-octanol were purchased from Merck. Additionally, 10 normal sodium hydroxide solutions were prepared by adding 0.75 mL of 10 N sodium hydroxide to 25 mL of methanol during stirring. Then 25 mL of n-Octanol and 0.75 mL of distilled water were added. This solution, a 0.15 N sodium hydroxide in alcohol, is suitable for use.

#### Calibration standards and standard samples for colorimetric part (standard TBC stock (1000 mg/kg))

This solution can be purchased if needed, otherwise, about 0.25 g of TBC should be dissolved in 300 mL of toluene and weight added to toluene. Equation (1) was used to calculate its exact concentration:

1 $$\frac{mg\,TBC}{{kg \;Toluene}} = \frac{{\left[ {{\text{g }}\;{\text{TBC*Purity }}\;{\text{of }}\;{\text{TBC}}} \right]{*}1000000}}{{{\text{g }}\;{\text{of }}\;{\text{Toluene}}}}$$

Five calibration standards with concentrations of 5, 10, 20, 30, and 40 mg/kg were prepared by diluting the stock solution with toluene.

Experiment: 5 mL of the sample was poured into a clean 100 mL Erlenmeyer flask. Then 100 µL of prepared sodium hydroxide alcoholic solution was added to the Erlenmeyer flask containing the sample and stirred well for one minute. Now 200 µL of methanol was added and stirred for another 15 to 30 s. A 1 cm cell was filled with a blank (sample to be tested), after cleaning the wall of the cell, the UV/Visible spectrophotometer zeroed with that at 490 nm. Then the cell was cleaned and dried, filled with the sample, put in the spectrophotometer, and read at the same wavelength. To increase the accuracy, this time should be the same as the time spent during calibration. The amount of TBC in the sample in terms of mg/kg is obtained from the calibration chart and is displayed by the device.

### Styrene without inhibitor for GC/FID standard

For this purpose, 600 mL of pure styrene was added into a separating funnel, and 200 mL of 1 N sodium hydroxide (NaOH) solution was added to it. The mixture was stirred well until the TBC in the styrene completely dissolved in the NaOH and separated. In this case, a red layer formed at the bottom of the funnel. This layer was removed and discarded. The process was repeated by adding another 200 mL of NaOH, and the bottom layer was again discarded. To ensure the absence of TBC, the washing was repeated with 100 mL of sodium chloride solution. Mixing NaOH with styrene may produce vapors, so while stirring the separating funnel, its valve was alternately opened and closed to release any accumulated vapors. The remaining styrene in the funnel was then washed three times with 200 mL of deionized water each time to remove residual NaOH and salts. The bottom water layer was removed after each wash. The pH of the final outlet water was checked to ensure it was approximately neutral (pH ≈ 7). TBC-free styrene was stored in a one-liter glass container. To ensure that styrene is free of TBC, some of it was removed and a TBC content was determined by colorimetric method. The GC/FID method requires minimal reagents, with no need for caustic solutions or organic solvents like methanol and n-octanol. This not only minimizes the risk of chemical exposure but also reduces the generation of hazardous waste. In contrast, the colorimetric method involves handling sodium hydroxide, methanol, and n-octanol, which pose significant health and environmental risks. Furthermore, the small injection volume (1 µL) in the GC/FID method reduces the likelihood of styrene vapor release, enhancing operator safety.

#### Preparation of TBC standard chemical solution to calibrate the GC/FID

**Fig. 1:**
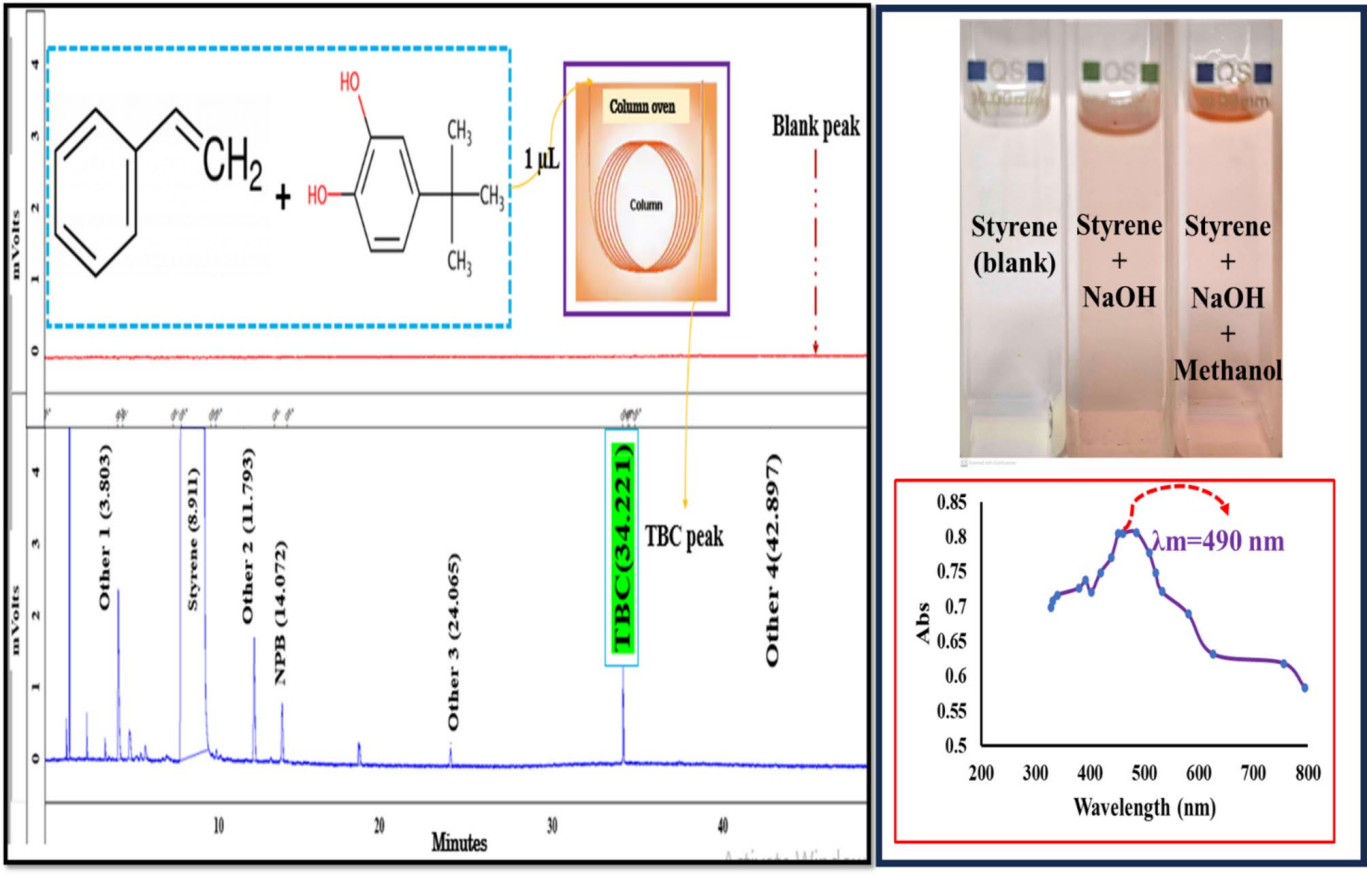
Comparative schematic representation of the two analytical methods.

Five clean 100 mL volumetric flasks were prepared. Concentrations of 5, 10, 20, 30, and 40 mg/kg were noted on it. 0.5, 1, 2, 3, and 4 mL of stock solution (2.1.1) were added to the flasks, respectively. And diluted by uninhibited styrene (2.2). The desired concentrations were obtained similar to the diluted standards of the colorimetric method. Calibration solutions are ready and usable. For testing, 2 mL of styrene containing TBC is poured into a special vial and 1 µL is injected into the GC through an autosampler connected to the chromatography device. All materials used in this test method must be reagent grade. But if other types of materials do not harm the accuracy of the test, they can be used. The general schematic of the two methods is illustrated below Fig. 1.

### Methods

To determine TBC chromatography in styrene, the GC/FID method was used. For the colorimetric determination of TBC in styrene, the ASTM D4590 test method was used using a Cary 50 model colorimetric spectrophotometer from (USA), with a wavelength of 200–800 nm, with small glass-shaped cells: d (optical path length of the cell) = 1 cm, scan speed = 4800 nm min^−1^, interval = 1 nm, Y Max = 1. In this method, by adding a caustic solution in methanol-octanol to the sample, a purple color is created, and this color intensity is measured using a spectrophotometer and compared with the calibration chart, and is considered as a standard for determining the amount of TBC. It should be noted that any other compound whose mixing with sodium hydroxide causes the production of color at the wavelength of 490 nm can cause problems for this method. If the nature and concentration of this interfering compound are known, it should be added to the calibration mixture.

### Conditions of the gas chromatography test method

According to past experiences^23–25^, changes were made in the conditions of the device for TBC analysis. A dimethylpolysiloxane column (CP-Sil 5 CB FS 25 m, 0.25 mm, 0.4 μm (CP 7709)) as a non-polar phase was used for TBC analysis. The initial column temperature was 50 °C for 10 min. Then the temperature was increased to 110 °C at a rate of 5 °C per min and then to 230 °C at a rate of 10 °C per min until the end of the analysis. The injector temperature was 260 °C. The detector temperature was 270 °C with nitrogen as the carrier gas at 19 psig. A flame ionization detector was also used using synthetic air (300 mL min^−1^), hydrogen (30 mL min^−1^), and a mixture of nitrogen (25 mL min^−1^). The injection volume was 1.0 µL and the split ratio: was 30. The internal normalized approach was selected for the calibration method of the instrument.

### Method validation

The signal-to-noise (s/n) method is used to determine LOD and LOQ concentrations in this study. 3: 1 is the accepted general signal-to-noise ratio for LOQ concentration calculations, while 10: 1 is considered necessary for LOQ concentration calculations. LOD levels should be able to identify the peaks. At least 3 should be used to determine the signal-to-noise ratio of the main peak produced by LOD solution injections. At least 10 should be used to determine the signal-to-noise ratio of the main peak produced by LOQ solution injections. Less than 10% of the chromatograms obtained from injections performed after LOQ% level determination should have a relative standard deviation (RSD%) of areas obtained from the main peak. The standard deviation of the linear response and the slope were used to calculate the LOD and LOQ using equations. where k is the slope of the calibration curve and σ is the standard deviation for the lowest standard concentration according to Eqs. (2) and (3), respectively^26^.

2 $${\text{LOD }} = { 3}.{3 }\sigma /{\text{k}}$$

3 $${\text{LOQ }} = { 1}0 \, \sigma /{\text{k}}$$

Reproducibility refers to a method’s ability to produce consistent results for multiple batches of the same sample. To test the reproducibility of the results in this study, five standard sample solutions were created. The PT sample was used to test the accuracy of the study’s method. For the PT sample, RSD should not exceed 10%. PT sample (IIS 23C07) with an average value of 6.919 mg/kg (by the results of the IIS (Institute for interlaboratory studies- Netherlands) output), 15 times with the TBC measurement colorimetric method (ASTM D4590), and the chromatography device, the method created in the Pars petrochemical Ethylbenzene/Styrene monomer (EB/SM) Laboratory, Asalouyeh, Bushehr, Iran were analyzed. Then, t-test and F-Test calculations were performed on the obtained results.

A calibration plot of absorbance and peak area versus TBC value was created manually using Excel and their linearity was checked. To determine if there is a statistically significant difference between different methods, one-way analysis of variance (ANOVA) tests was performed to compare the peak area and absorbance of the standard points obtained in the GC/FID and colorimetric apparatus and its linearity.

## Results and discussion

### TBC detection at a new peak in a GC/FID chromatogram

**Fig. 2:**
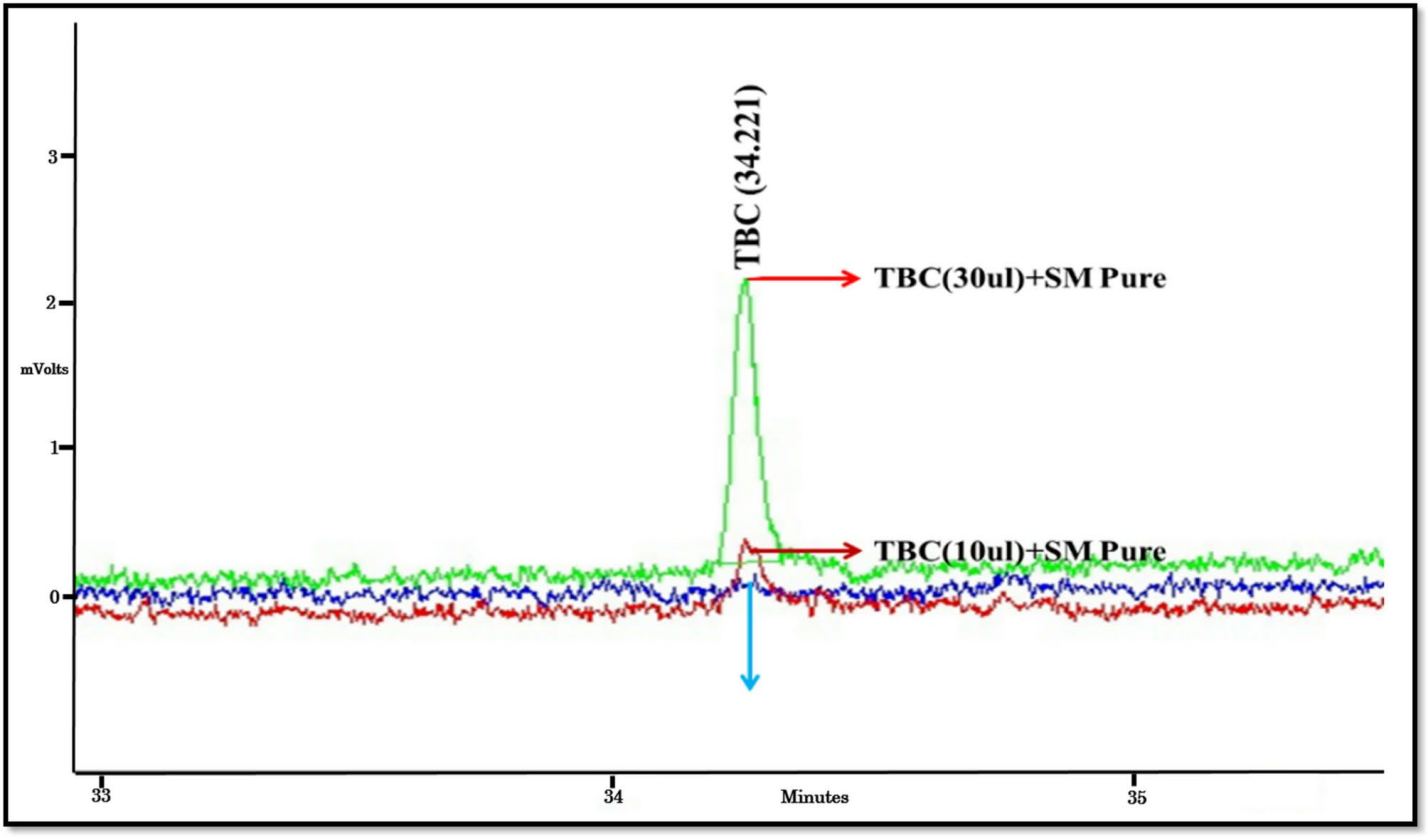
Identification of TBC peak.

TBC-free styrene was injected into the chromatographic device, and this injection was named as a control sample. Then, 10 μl of concentrated TBC standard sample was spiked with a specified amount of TBC-free styrene. The desired sample was injected into the machine and two chromatograms were compared. It was found that the added TBC sample appeared in approximately 34 min. To ensure the work, a larger amount of the concentrated sample with a higher concentration (30 μl) was spiked to a certain amount of TBC-free styrene and injected into the device, which confirmed the increase of the TBC peak in that area. (Fig. 2) TBC detected composition on the chromatogram.

### Preparation of GC/FID measurement method and its calibration with a prepared standard solution

The prepared standard samples were injected several times with the developed method under optimized temperature conditions. The previously identified TBC composition was calibrated with the concentrations in the standard by an internal normalization approach, and considering N-propylbenzene (NPB) as an internal standard (present in real samples), the relevant method was calibrated. NPB is a minor impurity that always exists in Styrene produced by Pars Petrochemical Company. The concentration of NPB has been used for internal normalization as an internal standard. Other styrene impurities could be considered internal if required. There is no need to add internal for sample analysis using the internal normalization approach. Real styrene samples and standard solutions were injected and analyzed by the method.

**Fig. 3:**
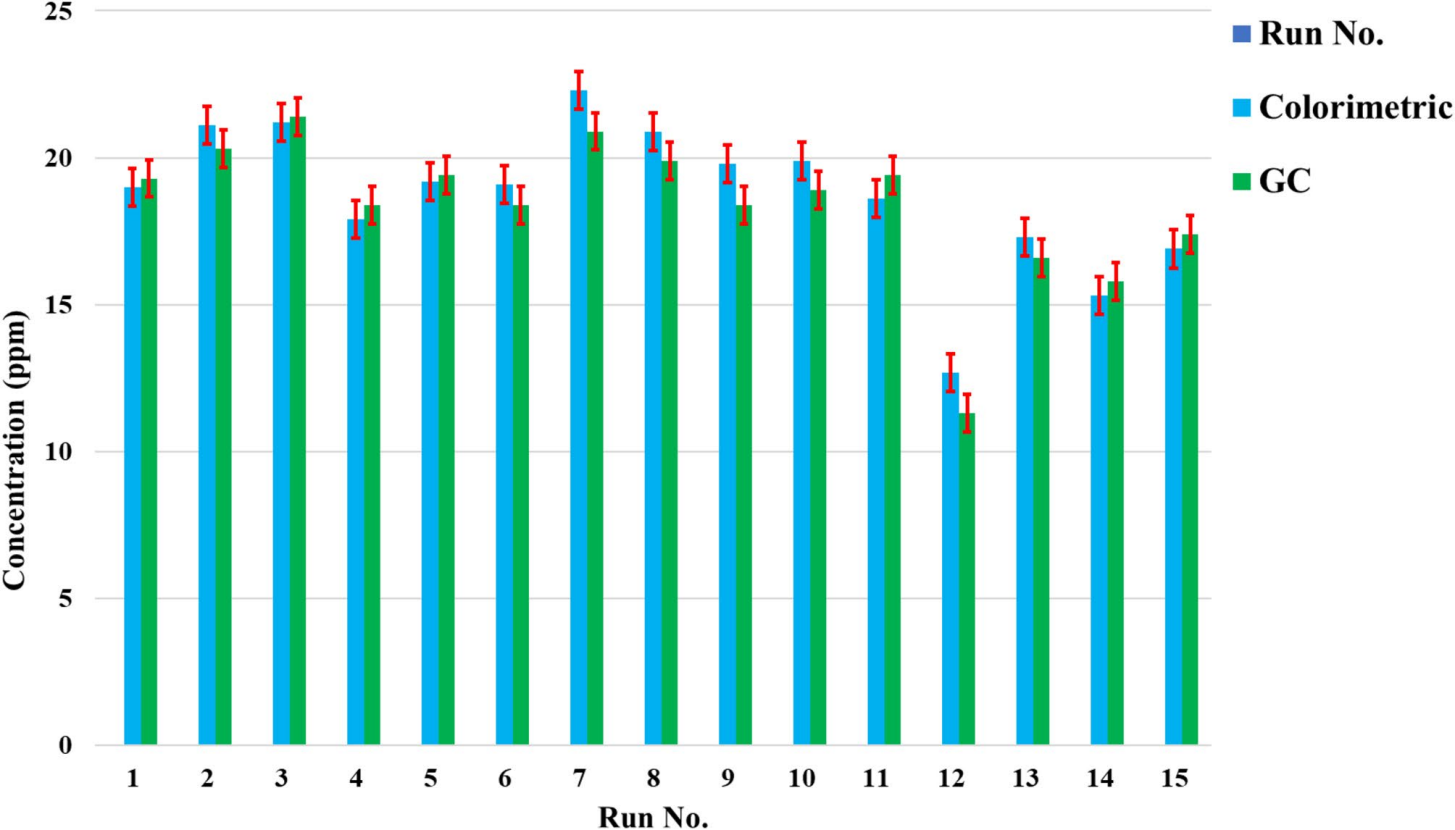
Comparing the results of TBC in styrene using two methods.

As shown in Fig. 3, during consecutive days for 15 days and with three repetitions, the styrene sample containing TBC (product sample of Pars Petrochemical Styrene Unit) was tested by both GC/FID and colorimetric methods. The results indicate no significant differences between the two methods, demonstrating that the GC/FID method is suitable for TBC determination. Further, in the validation method section, the comparison of these two methods has been discussed.

### Method validation

**Fig. 4:**
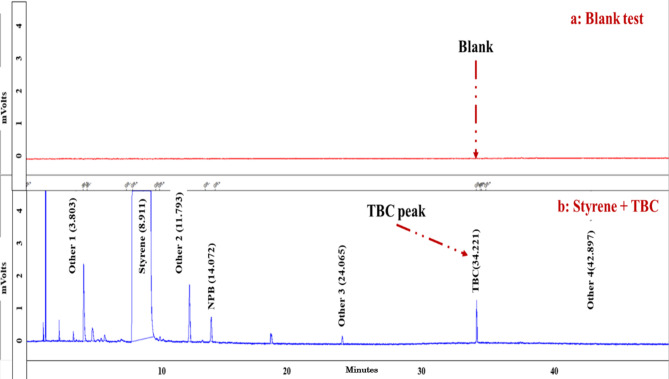
GC/FID comparison of a blank sample prepared from TBC-free styrene with a spiked sample: (**a**) Blank test; (**b**) Styrene + TBC.

This manuscript aimed to demonstrate a way to validate a correct and reliable approach for use in a device setting for TBC diagnosis. For this study, a standard sample was used to test multiple method validation parameters, including linearity, range, LOD/LOQ, and precision. In this research, the PT sample was also used to continue the investigation of two methods of TBC diagnosis. The specificity of the method was shown by comparing the chromatogram of a blank sample prepared from styrene without TBC with the spiked sample (Fig. 4). No interference in TBC peak appearance time was detected in that range, thus indicating high specificity for the method.

The method is specific to the property being measured. The desired method produces a high-quality reportable result thanks to the chosen performance characteristic. Additionally, the peak that appears suggests that neither the process nor the matrix component should cause any interference. The results of this research can be compared and confirmed with the results of the research of Winarsih et al.^27^.

**Fig. 5:**
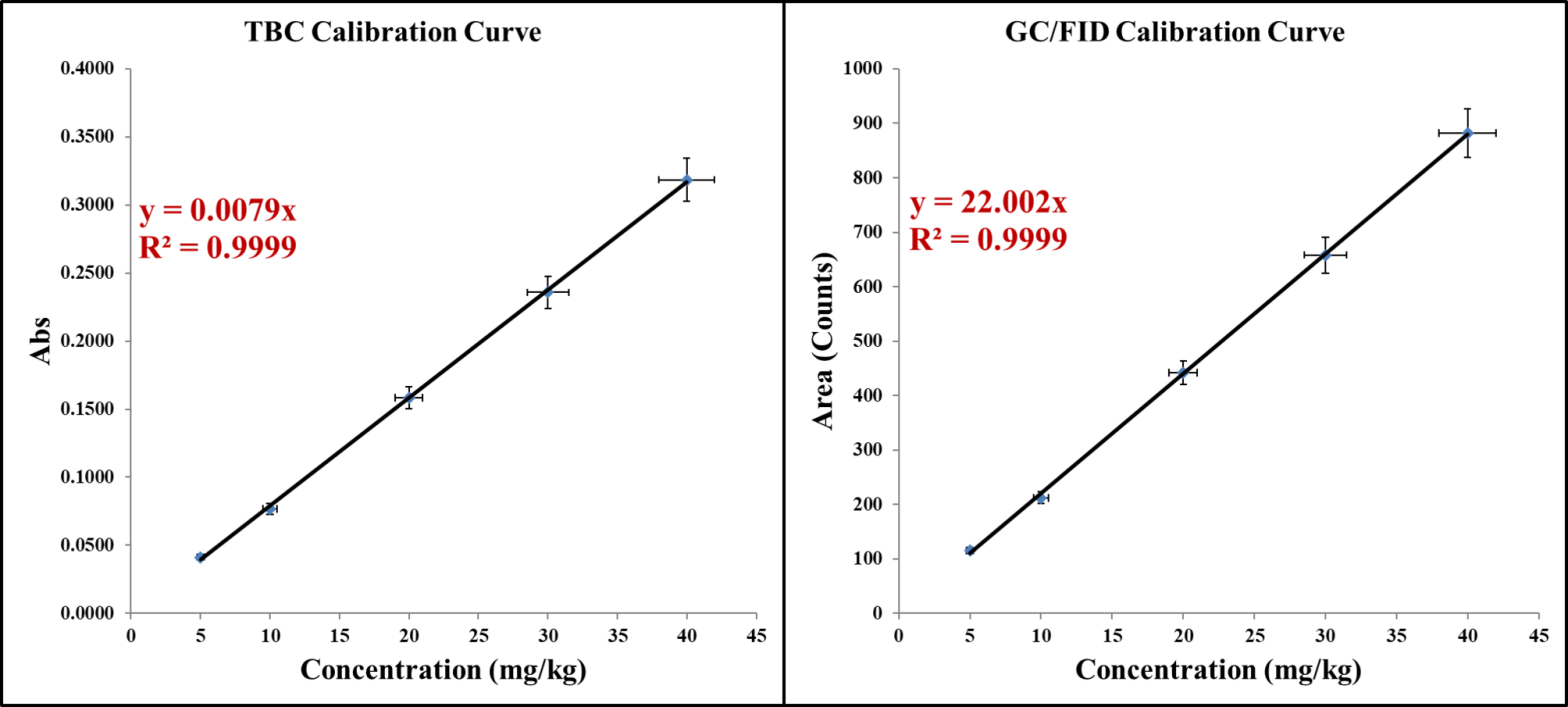
TBC calibration graph for linearity and range tests for both methods.

The accuracy of the GC/FID method was evaluated using the data obtained from the linear study and the TBC range of 5 to 40 mg/kg. A high recovery percentage was observed in the range above 99.0%. The linearity and amplitude were measured using standard samples with a concentration range of 5 to 40 mg/kg for both methods. In the analysis range of 5 to 40 mg/kg, the graph obtained showed an acceptable linearity. According to Fig. 5, the error bars in the graph represent the standard deviation of triplicate measurements of each TBC value. Additionally, all data points exhibited a standard deviation below 15.0%, indicating satisfactory reproducibility (Table 1).

**Table 1:** TBC accuracy results for two measurement methods.

TBC concentration (mg/kg)	Average peak area (n = 3) (Counts: U Volts*Sec)	Average absorption (n = 3)	Recovery relative- GC/FID (%)	Recovery relative- colorimetric (%)
5	115	0.0412	99.28	98.05
10	202	0.0767	99.03	99.45
20	514	0.1583	100	99.52
30	658	0.2358	99.89	100
40	882	0.3184	99.05	99.64

The standard deviation values were less than 15.0%, which indicates the consistency and reproducibility of the accuracy of the method. The results of this research are consistent with the method validation and new peak detection for the liquid chromatography-mass spectrometry method presented by Oyugi et al. and the compliance and regulatory considerations for the implementation of the multi-feature method presented by Gervais et al.^26,28^.

A statistically significant difference between different standards was done using (ANOVA) to compare the values of the sub-peak area and absorbance of standard points obtained by both methods and their linearity. Differences between analysts were considered significant if the ANOVA p-value was less than 0.05. Using the results obtained in (Table 2), the F value calculated using ANOVA is greater than the F critical value (F ˃ F critical). It means that there is a good linear relationship between X and Y values. On the other hand, the systematic error and slope of the calibration line were calculated for each round of the method using the Linnets function. The results showed that t is smaller than t critical (t ˂ t critical). This means that in these two methods, there is no systematic error in the width from the origin, and the continuation of t calculated is greater than t critical (t ˃ t critical), which means that the slope of the calibration line is significantly different from zero. All these results are summarized in Table 2.

**Table 2:** Calibration parameters for TBC for two measurement methods using ANOVA, and LINEST.

Parameters	GC/ FID (n = 3)	Colorimetric (n = 3)	Final result
t = a/Sa	− 0.24	− 0.30	t calculated < t critical
t critical	2.77	2.77	
t = b/Sb	161.31	144.73	t calculated˃ t critical
F	24,561.25	20,949.34	F calculated˃ F significance
F significance	1.36E−08	1.36E−08	

In addition to the cases that have been mentioned for the statistical confirmation of these two methods, it was further investigated using the PT sample that was held in October 2023 among 18 countries from 35 laboratories, and its results were confirmed (Pars Petrochemical EB/SM Laboratory was one of the participants that won satisfactory points for the number of times in a row- Lab Code: 7014). In this research, the same sample was used to compare the two methods. The result by the GC/FID method is about 6.9 mg/kg and in that case Z score is supposed to be near zero. Furthermore, the calculated t-value is lower than the critical t-value, and the calculated F-value is lower than the critical F-value, confirming that the accuracy and precision of the two methods are equivalent and acceptable, and it can be determined from the method created with Chromatography was used to measure TBC. The results of the calculations are given in Table 3.

**Table 3:** PT sample results for two methods of measuring TBC in styrene.

t-Test: two-sample assuming equal variances/F-Test two-sample for variances					
	GC/FID	Colorimetric		GC/FID	Colorimetric
Mean	6.98	7.24	Mean	6.98	7.24
Variance	0.53742857	0.51685714	Variance	0.53742857	0.516857143
Observations	15	15	Observations	15	15
Pooled variance	0.52714286		Df	14	14
Hypothesized mean difference	0		F	1.039801	
Df	28		P(F < = f) one-tail	0.47141087	
t Stat	− 0.98070822		F Critical one-tail	2.48372574	
P(T < = t) one-tail	0.16756752				
t Critical one-tail	1.70113093		Final result		
P(T < = t) two-tail	0.33513504		t calculated < t critical		
t Critical two-tail	2.04840714		F calculated < F critical		

On the other hand, the accuracy of each device and method was also checked with PT samples. In this way, the results obtained from the two methods were compared with the actual amount of TBC data extracted from the participating laboratories, and the t-test was performed on the data. According to the results of (Table 4), because the t calculated is lower than the t critical, it means that the accuracy of the device is confirmed.

**Table 4:** Determining the accuracy of two TBC measurement methods using PT samples.

Method	TBC average (mg/kg)—(n = 3)	Standard deviation	t calculated	t critical- (At the level of 95%-n = 15)	TBC average (mg/kg)—PT sample	Final result
GC- FID	6.98	0.79183	0.29836	2.14479	6.919	t calculated < t critical
Colorimetric	7.24	0.77653	1.53849	2.14479		

The proposed method was evaluated based on the analytical performance characteristics shown in (Table 5) to determine LOD/LOQ in the standard sample at three concentrations of 2, 2.5, and 3 mg/kg. The determination coefficient (R^2^) was higher than 0.99, which indicates a satisfactory linearity in the studied range. According to (Table 5), the LOD method showed 0.04–0.56 mg/kg and the LOQ 0.15–1.96 mg/kg for two chromatography and colorimetric methods, respectively.

**Table 5:** Determination of LOD/LOQ by GC/FID analysis on standard sample.

Method	Linear range—TBC (mg/kg)	Intercept	Slope	R^2^	LOD (mg/kg)	LOQ (mg/kg)
GC-FID	2 to 3	− 0.0005	0.4983	0.999	0.04	0.15
Colorimetric	2 to 3	− 0.4264	0.6593	0.991	0.56	1.96

No significant difference was found between the results of both methods of determining TBC, according to the statistical results obtained. Based on the validity of the method and practical application, this method has high efficiency and low cost. Most importantly, it showed that contamination from analysis is greatly reduced. It can be claimed that for the analysis of TBC in styrene samples, the chromatographic (GC/FID) method is a useful and effective alternative to the colorimetric method.

The statistical tests included signal-to-noise (S/N) analysis for LOD/LOQ, calibration linearity assessment using ANOVA, t-tests for systematic error and slope validation, F-tests for goodness of fit, and proficiency testing (PT) with Z-score analysis. These tests confirmed the accuracy, precision, and reproducibility of the GC/FID method, ensuring its reliability for TBC determination in styrene.

### Environmental implications

In the context of the identification and analysis of obtaining a variety of inhibitors that are injected into styrene to prevent polymerization, including TBC, it has already been reported in the relevant ASTM D4590, which is a colorimetric method. As mentioned, this method often creates contaminations that are harmful to technicians who work with styrene. Furthermore, the disposal of residual volumes after experiments poses significant environmental risks. Also, sometimes the leakage and more importantly the vapors created during the discharge in the hot season of the year and its effects are not compatible with the environment. In this research, for the first time, a method for determining TBC in styrene was reported, which can cover all the mentioned cases and expose the person to styrene less and have far less environmental pollution than the colorimetric method. The proposed method demonstrated robust performance in data quantification, thereby enhancing its potential applications. Since the data obtained from this proposed method can be compatible with the current model of TBC measurement in styrene. The developed method allows users and analysts to choose the most appropriate platform and approach for TBC determination in styrene and can provide informed decision-making for future inhibitor analysis in styrene. The results of this research are consistent with similar studies using chromatography instead of colorimetry^29–31^. The colorimetric method requires 5.3 mL of hazardous reagents per analysis, such as sodium hydroxide and methanol, leading to significant waste in industrial settings. For example, analyzing 100 samples daily generates 530 mL of waste, or 15.9 L monthly, posing environmental and safety risks due to improper disposal. In contrast, the GC/FID method uses only 1 µL of sample per injection, producing no hazardous waste and aligning with green laboratory practices. It also enhances operator safety by minimizing styrene handling and eliminating toxic reagents, reducing exposure to harmful vapors. While the colorimetric method is cost-effective for small-scale analyses, the GC/FID method is more suitable for industrial applications due to its minimal waste, improved safety, and long-term cost-effectiveness. By reducing chemical use and waste, GC/FID lowers environmental pollution and health risks from prolonged chemical exposure^32^.

## Conclusions

A new quantitative analytical method was developed for the determination of TBC in styrene using a chromatography device. It is evident from the obtained results that the main differences between chromatography and UV–Vis methods are as follows. In chromatography, TBC appears in a peak based on temperature conditions and is therefore determined in an unchanged form. UV–Vis spectrophotometry is used to absorb reaction products with alkaline hydroxide as a reagent and identifier to determine TBC. Therefore, no significant differences were observed in the results of both methods to determine TBC. The real styrene sample was tested by the developed method several times on different days and no significant difference with the colorimetric result. This method demonstrated high efficiency and simplicity, as confirmed by rigorous method validation and practical implementation. In conclusion, while UV–Vis spectroscopy is an effective method, gas chromatography is preferable for analyzing large numbers of samples with high accuracy and minimal chemical exposure. On the other hand, due to the ease of performing the test in a very small volume (injection volume: 1 µL—injection with an autosampler) in the GC/FID method compared to the colorimetric method, the contamination caused by the TBC test is greatly reduced. Due to the possibility of styrene being carcinogenic, the risk of exposure to this substance and its vapors is greatly reduced by the GC/FID method. Also, there is less chance for human error and mistakes. In conclusion, the GC/FID method provides a safer alternative to the colorimetric method for TBC determination in styrene. By minimizing operator exposure to hazardous chemicals and styrene vapors, reducing contamination risks, and aligning with green laboratory practices, the GC/FID method ensures a safer working environment. These safety advantages, combined with its high accuracy, simplicity, and efficiency, make the GC/FID method an ideal choice for routine industrial analysis.

## Data Availability

The datasets generated and/or analyzed during the current study are available on request from the corresponding author.

## Abbreviations

TBC: 4-Tert-butylpyrocatechol
ASTM: American Society for Testing and Materials
ANOVA: Analysis of variance
EB/SM: Ethylbenzene/Styrene monomer
FID: Flame ionization detector
GC: Gas chromatography
IIS: Institute for interlaboratory studies
LOD: Limit of detection
LOQ: Limit of quantification
NPB: *N*-Propyl benzene
PT: Proficiency test
RSD: Relative standard deviation
UV–Vis: Ultraviolet–visible spectrophotometry
VOCs: Volatile organic compounds
